# Optimizing ultrathin Ag films for high performance oxide-metal-oxide flexible transparent electrodes through surface energy modulation and template-stripping procedures

**DOI:** 10.1038/srep44576

**Published:** 2017-03-14

**Authors:** Xi Yang, Pingqi Gao, Zhenhai Yang, Juye Zhu, Feng Huang, Jichun Ye

**Affiliations:** 1 Ningbo Institute of Material Technology and Engineering, Chinese Academy of Sciences, Ningbo 315201, China

## Abstract

Among new flexible transparent conductive electrode (TCE) candidates, ultrathin Ag film (UTAF) is attractive for its extremely low resistance and relatively high transparency. However, the performances of UTAF based TCEs critically depend on the threshold thickness for growth of continuous Ag films and the film morphologies. Here, we demonstrate that these two parameters could be strongly altered through the modulation of substrate surface energy. By minimizing the surface energy difference between the Ag film and substrate, a 9 nm UTAF with a sheet resistance down to 6.9 Ω sq^−1^ can be obtained using an electron-beam evaporation process. The resultant UTAF is completely continuous and exhibits smoother morphologies and smaller optical absorbances in comparison to the counterpart of granular-type Ag film at the same thickness without surface modulation. Template-stripping procedure is further developed to transfer the UTAFs to flexible polymer matrixes and construct Al_2_O_3_/Ag/MoO_x_ (AAM) electrodes with excellent surface morphology as well as optical and electronic characteristics, including a root-mean-square roughness below 0.21 nm, a transparency up to 93.85% at 550 nm and a sheet resistance as low as 7.39 Ω sq^−1^. These AAM based electrodes also show superiority in mechanical robustness, thermal oxidation stability and shape memory property.

Transparent conductive electrodes (TCEs) with high conductivity and transparency are essential elements for the advanced optoelectronic devices, such as touchscreen panels (TSPs), solar cells (SCs), and organic light emitting diodes (OLEDs)^1^,^2^,^3^,^4^. To date, conductive films including conducting metal oxides, graphene, carbon nanotubes (CNTs), conducting polymers, metal nanowires, metal mesh, ultrathin metal films (UTMFs), and hybrids of them have been reported as potential candidates to replace traditionally indium tin oxide (ITO), which is limited by its fragility, high refractive index, limited indium supplies, and high processing temperature^5^,^6^,^7^,^8^,^9^,^10^,^11^,^12^. Among these alternatives, ultrathin Ag films (UTAFs) have emerged as a very promising candidate due to their excellent electrical conductivity, low optical loss in the visible range, excellent mechanical flexibility, and mature mass production. However, simultaneously achieving low electrical resistance and high optical transmittance in UTAFs remains challenging due to the poor film homogeneity and continuity when the film thickness is below 10 nm. To achieve optically thin and electrically thick UTAFs, the previous investigations were prone to improve the wettability of the metals and thus to reduce percolation thickness by controlling deposition rate, substrate temperature, seed layers and nitrogen or oxygen doping^13^,^14^,^15^,^16^,^17^,^18^,^19^,^20^,^21^,^22^,^23^,^24^,^25^,^26^. Moreover, there is another big challenge in fabricating UTAFs directly on flexible substrates due to the incompatibility issues between fabrication conditions and thermal, mechanical, and chemical properties of these substrates^27^,^28^. The rough surface of as-deposited metallic film on flexible substrate will not only reduce optical transparency *via* light scattering and plasmonic resonances induced parasitic absorption but also limit the electrical conductivity due to the electron scattering at grain boundaries^29^,^30^,^31^,^32^. To overcome these challenges, transfer printing methods, which use conventional substrates for the fabrication of films and then deliver the films onto flexible substrates, can be used to transfer the natural flatness of substrate to metal films and result in metal surfaces with topographic features in Angstrom dimensions^33^,^34^,^35^,^36^. Nevertheless, the issues of strong reflection of incident light as well as oxidation and corrosion of metal remains, which makes it difficult for UTAFs to be implemented for practical use in flexible optoelectronics.

Here, we demonstrate that a flexible tri-layer TCE composed of an ultrathin and ultrasmooth Ag film sandwiched between aluminium oxide (Al_2_O_3_) and molybdenum trioxide (MoO_x_) can be fabricated by surface energy modulation combined with the template-stripping process. By minimizing the surface energy difference between Ag film and silicon substrate, a completely continuous Ag film is produced at an ultralow thickness down to ~9 nm. After the deposition of Al_2_O_3_ layer as a top anti-reflective and protective coating, the Al_2_O_3_/Ag bilayer film is transferred to a flexible polymer matrix through the template-stripping process. The newly exposed smooth Ag surface which originally contact with Si substrate exhibits an ultralow surface roughness down to the sub-nanometer scale which is beneficial for improving the surface features of the subsequent MoO_x_ layers. The MoO_x_ layer is then deposited as a bottom anti-reflective layer, and a highly uniform and smooth Al_2_O_3_/Ag/MoO_x_ (AAM) film is finally achieved. By adjusting the thickness of each layer, the optimized AAM film exhibits a low sheet resistance (*R*_s_) of ~7.39 Ω sq^−1^ and a high optical transmittance (*T*) of ~93.85% at 550 nm. In addition, the AAM based TCE also shown superiorities in mechanical properties, thermal oxidation stability and shape memory property.

## Results

### Fabrication of ultrathin Ag film

Contrary to the extraordinary electrical conductivity of bulk metal, thin metallic films often exhibit relatively high *R*_s_ due to the Volmer-Weber (island) growth mode when the film thickness is comparable to the electron mean free path (MFP), and even become an insulator when the thickness is below the percolation length^31^. To suppress the growth of large metal islands during film deposition, an abrupt morphological transition from granular, discontinuous three-dimensional (3D) films to smooth, continuous two-dimensional (2D) films is facilitated by reducing the energy difference between Ag film and substrate^37^:


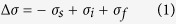


where *∆σ* is the energy difference between the metal film and substrate, *σ*_s_ is the surface free energy of the substrate, *σ*_i_ is the free energy of the interface between the substrate and film, *σ*_f_ is the free energy of the film and can be negligible during this early stage but increases linearly with increasing film thickness. With the decreasing of *∆σ*, the Ag atoms become more strongly bound to the substrate than to each other. By this means, Frank-van der Merwe (layer) growth mode can be achieved, making the formation of planar sheets possible.

To experimentally study the influence of *∆σ* on the growth of UTAFs, we deposited Ag films with different thicknesses on silicon substrates that were pre-treated by HF, UV and polyethyleneimine (PEI), respectively. The *σ*_*s*_ is investigated by measuring the contact angle (CA) of the pre-treated substrates in a sessile water drop experiment (Fig. S1). The hydrophobic substrate treated by HF exhibits a low *σ*_*s*_ with non-wettable CA of 75.14° due to the existence of non-polar hydrogen bond (-H) terminations. In contrast, the UV and PEI treated surface exhibit hydrophilic properties with wettable CA of 0˚ and 5.86˚, respectively. The *σ*_*s*_ in this case is high due to the presence of polar functional groups, including hydroxyl groups (-OH) on the silicon surface and amines (-NH_*x*_) from the PEI^38^. Subsequently, the *σ*_*i*_between the Ag film and the substrate is investigated by high-resolution N 1 s and Ag 3d X-ray photoelectron spectroscopy (XPS) spectra for samples of PEI treated Si substrates (PEI samples), Ag deposited on Si substrates (bare Ag samples) and Ag deposited on the Si substrates with PEI treatment (Ag/PEI samples). Compared to those of the bare Ag and PEI samples, Ag/PEI sample exhibits a negative chemical shift at the Ag 3d core level and a positive chemical shift at the N 1 s core level, while the XPS spectra of C 1 s core level and O 1 s core level remain unchanged (Fig. S2). These chemical shifts indicate that the functional -NH_*x*_ groups of PEI offer the unshared electron pairs to the Ag atoms to form coordinate covalent bonds and result in the weakest *σ*_*i*_ among the three kinds of samples^11^,^38^,^39^.

**Figure 1:**
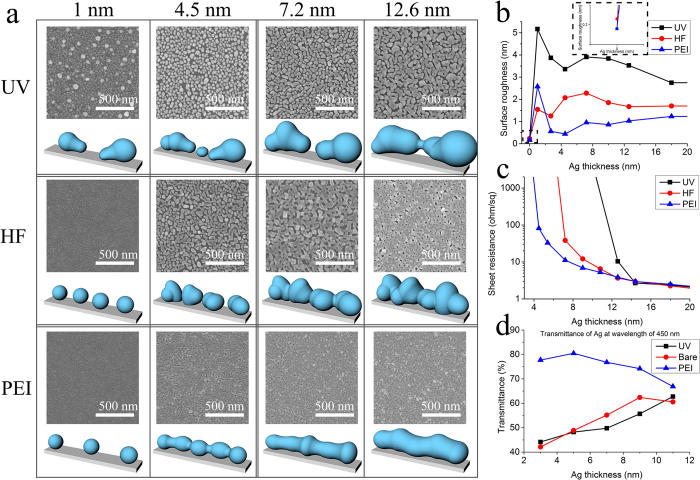
Performance of Ag films. (**a**) Scanning electron microscopy images showing morphological differences between 1-, 4.5-, 7.2- and 12.6-nm-thick Ag films deposited on silicon substrates with pre-treatments by UV, HF and PEI, respectively. Scale bar, 500 nm. Conceptual diagrams showing the cluster coalescence mechanism of Ag film on each stage. (**b**) Root-mean-square surface roughness of Ag films on different substrates varying with Ag thickness. Average surface roughness was determined from the measurement of at least three different scan domains for each sample. The inset shows the RMS of three substrates. (**c**) Variations in sheet resistance of Ag films on different silicon substrates as a function of Ag thickness. (**d**) Transmittance of Ag film on UV treated, bare, and PEI treated quartz substrates, respectively, at the wavelength of 450 nm.

Figure 1a schematically illustrates the cluster evolution mechanism during early growth stages of Ag on silicon substrates that were pre-treated by HF, UV and PEI, respectively. The Ag thickness-dependent *R*_*s*_ and root-mean-square (RMS) roughness on different substrates are also shown in Fig. 1b,c. In the initial nucleation stage, as represented by the film with 1 nm in thickness, small island-like clusters are easily formed on the low *σ*_*s*_substrate. The clusters contact with the substrate by point style due to the de-wetting behavior. While for the substrates with high *σ*_*s*_, the driving force of the wetting behavior is increased to maintain the thermodynamic stability, leading to a gradual change of the contact from point to facet. After the initial stage, condensed atoms established permanent residences on the substrate, the following deposited atoms develop a uniform distribution of highly mobile clusters with unfilled channels in between. For the same deposition condition (the same number of deposited atoms), atoms on high *σ*_s_ substrate will cover more area than that on low *σ*_s_ substrate to maintain a relatively low system energy per unit area. Thus, Ag deposited on HF treated substrate exhibits a short channel distance and a large grain density which results a lowest RMS roughness among the three samples. In this stage, the RMS roughness of the film is increasing along with the deposition, while the *R*_*s*_ is infinite because the film is still made up primarily of discrete islands.

In the next coalescence stage, the discrete islands begin to merge with each other after rapidly saturations of the island density. For the films deposited on the high *σ*_*i*_ substrates (HF and UV treated), Ag atoms tend to coalesce with each other under a strong cohesive force between adatom to adatom but a weak adhesive force between adatom to substrate. Such atoms coalescences and unstable nuclei reduce the island density and result in a rough 3D Ag islands with local denuding of the substrate where further nucleation can occur. While for the low *σ*_*i*_ substrate (PEI treated), Ag atoms are immobilized and fixed on the substrate via coordination-bond interactions. The Ag-substrate interaction is stronger than the Ag-Ag interaction and the extension of small and stable nucleus occurs overwhelmingly, leading to the formation of a 2D Ag layer with a large grain density, small grain size, smooth surface, and low percolation threshold thickness. With further deposition, electrical conduction channels and first percolation paths of the film are formed. The percolation threshold thickness thicknesses for the substrates treated by UV, HF and PEI are therefore of 12.6 nm, 7.2 nm and 4.5 nm, respectively, fully demonstrating a *σ*_*i*_ dependent behavior. Beyond this critical thickness, the channels between the islands become smaller and more regular, and finally a uniform Ag layer is formed. In this stage, both the RMS roughness and *R*_s_ of the films are decreasing along with the deposition.

In the last stage, which we call it the film thickening stage, the whole surface of substrates has been covered by the Ag film. The *σ*_i_ and *σ*_s_ are negligible and the *∆σ* is dominated by the *σ*_f_, which increases linearly with increasing film thickness. The RMS roughness remains stable in this stage for all the three samples. The *R*_s_ further decreases with the increasing of film thickness, ultimately attaining a value often approaching that of the bulk.

In addition, the differences in *R*_s_ can be reflected in the morphological features of the films. As shown in the height (Fig. S3) and corresponding phase contrast (Fig. S4) images of Ag films growth on the different substrates. The 9 nm Ag films on substrates treated by the UV and HF have large grainy structures with RMS roughness of 3.91 nm and 2.07 nm, respectively. In contrast, the films on substrates treated by PEI reveal a relatively smooth and homogeneous surface morphology with RMS roughness of only 0.84 nm with an unobservable phase contrast. As shown in Fig. 1c, due to the strong electron scattering from the rough film interfaces and grain boundaries, the Ag film without PEI treatment show poor electrical conductivity until its thickness approach around 14 nm. As a result, the 9 nm Ag film deposited on the PEI treated substrate delivers a remarkably low *R*_s_ of 6.9 Ω sq^−1^, which is much lower than that of the ITO layer (20–50 Ω sq^−1^).

Moreover, to optically demonstrate the effect of surface energy modulation on growth of Ag, spectral transmissions of Ag films deposited on bare, UV and PEI treated quartz substrates are also measured. As we known, the transmittance of Ag film is always increasing with the reduction of the film thickness in the ultraviolet and near-infrared spectral ranges (Fig. S5) due to the polycrystalline nature^33^. However, in the wavelength range around 450 nm (Fig. 1d), which is the surface plasmon resonance wavelength center of Ag, an evident reduction of the transmittance is observed when the thickness of Ag films on bare, UV and PEI treated substrates is decreased to 10.7 nm, 11.6 nm and 4.2 nm, respectively. These transmittance reductions are mainly due to the excitation of surface plasmons^32^,^40^, and become more serious with the further decreasing of the film thickness. The results indicate that films with such low thicknesses are still composed of discontinuous island clusters, and the voids inside the volume of the metal film give rise to a non-uniform distribution of matter and also a large surface roughness.

### Fabrication and performance of Al_2_O_3_/Ag/MoO_x_ based transparent conductive electrode

**Figure 2:**
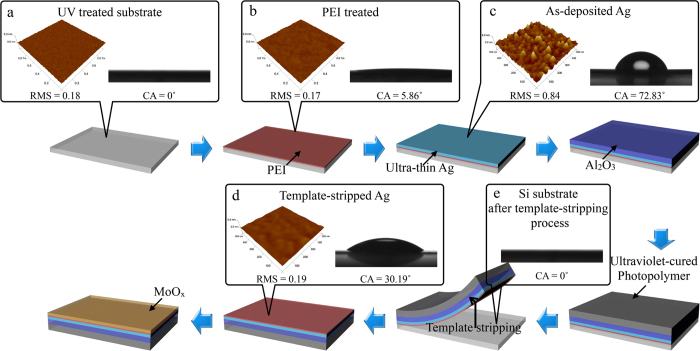
Schematic illustration of the process for fabricating the AAM based TEs. AFM images and photographs of the measured contact angle of (**a**) UV treated substrate, (**b**) PEI treated substrate, (**c**) As-deposited Ag film and (**d**) Template-stripped Ag film. (**e**) Si substrate after the template-stripping process, the CA indicate that the PEI was peeled off together with Ag film after the template-stripping process.

Although continuous Ag film with the thickness down to 9 nm can be fabricated by reducing the energy difference between Ag film and hard silicon substrate, the overall transmittance of the bare Ag film is still worse than that of the ITO layer due to the strong reflection of the incident light at the interfaces of air/Ag and Ag/substrate. To address this issue and transfer the film to the flexible substrate, we employed a template-stripping process to fabricate a flexible UTAF based TCE with a structure of Ag layer sandwiched in two different metal oxides, denoted as ‘Al_2_O_3_/Ag/MoO_x_ (AAM)’, as illustrated in Fig. 2. Firstly, UTAF is fabricated on the PEI treated substrates, followed by the deposition of a Al_2_O_3_ layer as a top anti-reflective coating as well as a water corrosion and atomic oxygen protector^41^,^42^. Then, a thin layer of commercial Norland Optical Adhesive (NOA 63), which is used as an encapsulating polymer matrix and flexible substrate for its excellent optical transparency in the wide spectral range and superior mechanical flexibility, is slot-die or spin coated onto the Al_2_O_3_ layer and then exposed to an ultraviolet light source^43^. Owing to the better adhesion of Al_2_O_3_ to cured NOA 63 (also the adhesion of Al_2_O_3_ film to Ag) than that to the substrate, Al_2_O_3_/Ag stack can be transferred onto the NOA 63 after the stripping of the cured film. Ultimately, MoO_x_ layer was deposited on the stripped film as a bottom anti-reflective coating to furtherly improve light transmission due to its high refractive index (Fig. S6). Note that the deposition of metal and metal-oxide film is mature mass production via processes such as sputtering, a further scale up of the flexible AAM based TCEs with the combination of continuous roll-to-roll processes is quite practicable (Fig. S7).

The surface morphologies of both as-deposited Ag film and stripped Ag film before and after MoO_x_ coating are investigated by AFM, as shown in Fig. 2c,d. The roughness of as-deposited Ag film on PEI treated substrate is 0.84 nm, while the one at the stripped Ag film is only around 0.19 nm (that is stemmed from the flat Si substrate of ~0.18 nm). This improvement will be inherited by the subsequently deposited MoO_x_ film with a RMS roughness of 0.21 nm, as indicated in Fig. S8. To further demonstrate the improvement of interface between the Ag film and the MoO_x_ layer, the CA of both as-deposited and stripped Ag film are shown in Fig. 2d. The CA of stripped film (30.19°), which is much smaller than the as-deposited one (72.83˚), results in a large contact area and thus a better adhesion to the subsequent MoO_x_ film. To estimate the adhesion of NOA 63 layer to stripped films, scraping tests are conducted using the blade of a utility knife (Fig. S9). No obvious changes in the surface of stripped film is observed after testing, while the as-deposited Ag can be easily removed by blade or even human hands due to the lattice mismatch between film and substrate. The tests are highly qualitative but allow us to avoid films with poor adhesion upon which to build devices.

**Figure 3:**
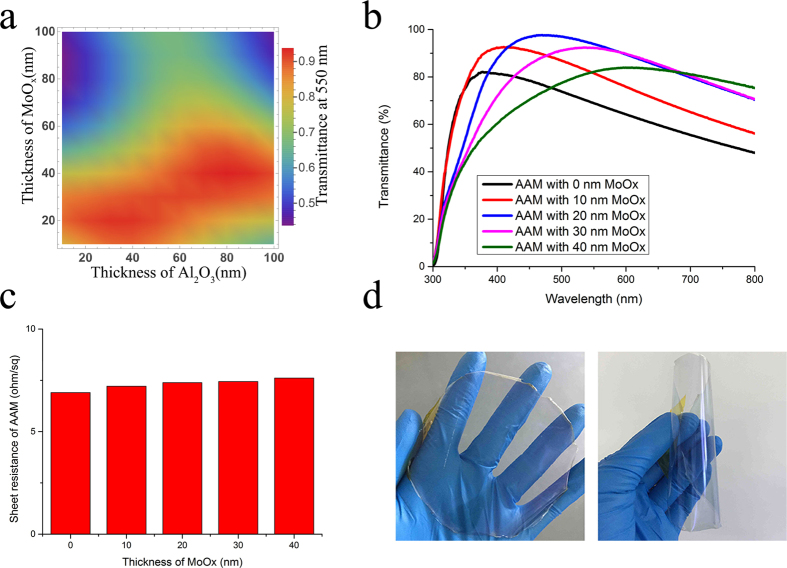
Performance of AAM based TEs. (**a**) Calculated transmittance of AAM film at the wavelength of 550 nm as a function of the thickness of MoO_x_ and Al_2_O_3_ layers. The thickness of Ag is fixed at 9 nm. (**b**) Transmittance spectra and (**c**) sheet resistance of AAM film with a fixed Al_2_O_3_ thickness at 9 nm and various MoO_x_ thicknesses in experiment. (**d**) Optical photographs of flexible AAM based TCEs peeled off from 4-in wafer size silicon by template-stripping process.

The optical and electrical properties of the AAM structures depend on the thickness of each layer. A 9-nm-thick Ag layer is introduced to ensure a low sheet resistance, while the thicknesses of the Al_2_O_3_ and MoO_x_ layers are varied in order to optimize the optical transmittance of the AAM films. Figure 3a shows the simulated transmittance of AAM films at wavelength of 550 nm with various Al_2_O_3_ and MoO_x_ thicknesses. It can be observed that the transmittances of the AAM films can be precisely modulated by simply changing the thickness of the Al_2_O_3_ and MoO_x_ layers. The measured transmittance spectra of AAM films with different MoO_x_ thicknesses are in good agreement with these calculated results as shown in Fig. 3b. For a single layer of Ag, the low transmittance is associated with the parasitic absorption and the high reflectance. After being sandwiched between the Al_2_O_3_ and MoO_x_ layers, both the parasitic absorption and the reflectance in the whole visible wavelength range are reduced due to the destructive interference of the incident light at the interfaces. The best AAM (40/9/20) film presents a transmittance of 93.85% at 550 nm and a maximum transmittance of 97.68% at 470 nm in our experiment. Due to the smooth surface and low light scattering, the AAM films also exhibit relatively uniform haze (~2.5%) in the visible light wavelengths (Fig. S10). This high transmittance and low haze for the AAM films are favorable for some special applications such as in displays and smart windows^44^. Moreover, the effect of MoO_x_ thickness on the electrical performance of AAM film is also evaluated. Although the *R*_*s*_ of MoO_x_ layer itself is much higher than that of Ag, there is only a little bit increase in *R*_*s*_ of the AAM film when the MoO_x_ thickness is varying from 0 nm to 40 nm due to the metallic properties of the MoO_x,_ as shown in Fig. 3c^45^. The relationship between transmittance and sheet resistance in AAM based TCEs as well as in many other TCEs can been analyzed through a commonly used figure of merit (FoM) by using the following widely accepted expression:


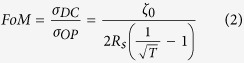


where *σ*_DC_ is the DC conductivity and *σ*_OP_ is the optical conductivity of the film, *T* is the optical transmittance at a wavelength of 550 nm, *R*_s_ is the sheet resistance, *ξ*_0_ = 120*π* is the impedance of free space. The higher FoM, the lower the sheet resistance at a given transmittance. The FoM value obtained from the single layer Ag film is 145, lower than that of ITO (175). However, the AAM based TCEs show a significant improvement in FoM. This means that the enhancement of optical transparency over-compensates for the reduction in electrical conductivity, resulting in an overall improvement in FoM. Figure 3d exhibits photographs of 4-in wafer size optimized AAM based TCEs with the FoM value of 791. Details on the optical and electrical parameters of transmittance, sheet resistance, and FoM obtained from ITO, Ag, AAM and other OMO film based TCEs in recent studies are summarized in Table 1.

**Figure 4:**
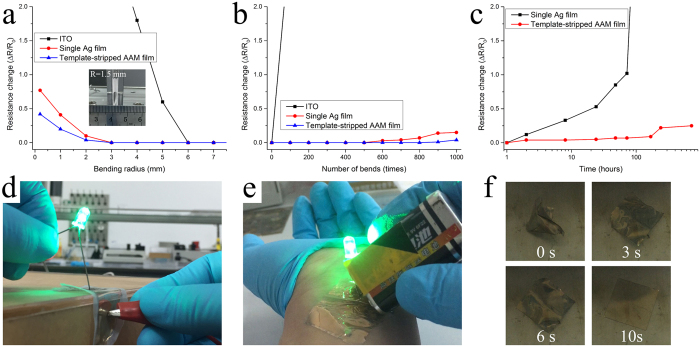
Flexibility and stability of AAM based TEs. (**a**) Relative change in the resistances of the ITO on PET, single Ag layer on PET, and stripped AAM film as a function of bending radius. The inset shows a photograph of a folded AAM based TCE with a bending radius of 1.5 mm set for the bending test. (**b**) Relative changes in the resistances of the ITO, single Ag, and stripped AAM film as a function of the number of bending cycles at the bending radius of 3 mm. (**c**) Relative changes in the resistance of the single Ag and stripped AAM film as a function of the exposing time up to 1000 hours. Photographs of blue LED lamps mounted on flexible AAM based TCE on (**d**) sharp wooded edge and (**e**) human skin. (**f**) A series of photographs showing the shape memory property of flexible AAM based TCE after baked at 100 °C.

Besides the excellent surface morphology, conductivity and transparency, the stability of AAM film under mechanical stress has a significant effect on the performance of flexible devices. Figure 4a shows how the *R*_s_ of commercially ITO film on PET substrate, Ag film on PET substrate, and stripped AAM film on NOA 63 substrate change under high compressive stresses conditions. The relative changes of *R*_s_ can be expressed as a normalized ∆*R*/*R*_0_ where ∆*R* is the actual change in the sheet resistance after bending and *R*_0_ is the initial sheet resistance. For bare Ag film on PET substrate, no obvious change in the ∆*R*/*R*_0_ is observed even after a 3-mm bending radius is applied. In contrast, the ∆*R*/*R*_0_ of the flexible ITO electrode dramatically increases to 0.6 when the bending radius approaching to 5 mm, which is due to the formation and propagation of microscopic cracks in a direction perpendicular to that of the compressive loaded. It is noteworthy that the ∆*R*/*R*_0_ of the stripped AAM film remains nearly a constant, even when the bending radius is pushed up to 200 μm. Moreover, we find that the ∆*R*/*R*_0_ of the stripped AAM film can be kept at nearly a constant after 1000 bending cycles at a 3-mm radius of curvature, whereas the resistance of the ITO electrode drastically increased (Fig. 4b). This is mainly attributed to the strong bonding between the NOA 63 matrix and AAM film, which could prevent the slides from occurring at the interface under the extreme bending. Furthermore, the stability in sheet-resistance of the AAM film is investigated as a function of time by exposing it to ambient environment (Fig. 4c). The stripped AAM film exhibits a slightly increased ∆*R*/*R*_0_ after exposing to ambient environment for 100 h. For comparison, the ∆*R*/*R*_0_ of the single Ag layer under the same condition dramatically increases to 2.0, which illustrates that the AAM structure can effectively protect the sandwiched Ag layer from oxygen gas and moisture. Figure 4d,e demonstrate the flexible AAM based TCEs can be successfully attached to sharp wooden edge and human skin using a double sided transparent scotch tape, enabling the conformal contacts with various material surfaces, and therefore making the surfaces conductive without any surface treatment. Additionally, stripped AAM films also show excellent shape memory property due to the freezing and activation of the long-range motion of polymer chain segments^43^. As shown in Fig. 4f, the deformed electrodes can reversibly relax back to their original shapes when be heated above glass transition temperature for ten seconds. Due to these excellent mechanical flexibility and thermal oxidation stability, the stripped AAM film has a great potential to establish its applications as a transparent electrode platform in the flexible electronic devices.

## Discussion

In conclusion, by the modulation of surface energy difference in combination with the template-stripping process, we demonstrate high-performance, flexible ITO-free TCEs composed of UTAFs sandwiched in between Al_2_O_3_ and MoO_x_ layers. The pre-treatment of the substrate by PEI produces a minimized surface energy difference between Ag film and substrate, and the resultant UTAF exhibits a completely continuous morphology with *R*_s_ of 6.9 Ω sq^−1^at the thickness of only 9 nm. Compared to a single layer of Ag film, AAM based TCE shows a low *R*_s_ of 7.39 Ω sq^−1^and a high transparency of 93.85% at the wavelength of 550 nm due to the reduced reflection of the incident light at both interfaces of substrate/Ag and Ag/air. Most importantly, the template-stripping process offers the AAM based TCEs superiority surface morphology (with RMS roughness of only 0.19 nm) as well as excellent mechanical flexibility and thermal oxidation stability. We anticipate that the technique presented here will potentially deliver high performance TCEs with full functions for high-end flexible electronic applications.

## Methods

### Electrode fabrication

Silicon substrate was cleaned with acetone, alcohol, and deionized water. PEI (Sigma-Aldrich, 50 wt% in H_2_O) was diluted in deionized water to create a 0.3 wt% aqueous solution. The pre-cleaned silicon substrate was treated with ultraviolet-ozone. The PEI solution was spin-coated onto the silicon at 5,000 r.p.m. and air dried at 100 °C for 20 min. The sample was then loaded into an electron beam chamber (Xinnan-Tech ZZS-500). 9 nm Ag film and 40 nm Al_2_O_3_ film were successively deposited on the silicon at a rate of 1 Å/s at a base pressure of 6 × 10^−4^ Pa. Then, a photopolymer (NOA63, Norland) film was spin-coated at 1000 rpm for 20 s and then exposed to an ultraviolet light source for 5 min. The power of the light source is 125 W. A pair of pre-cleaned tweezers was used to prise up an edge of the cured photopolymer film from the silicon, then the film was slightly peeled off from one edge toward another and immediately loaded into the electron beam chamber again. Finally, MoO_x_ film was deposited on the stripped Ag film and a tri-layer TE composed of an ultrathin film of Ag sandwiched in between Al_2_O_3_ and MoO_x_ layers was obtained.

### Characterization

The electrical properties of the films were measured using a four-point probe system (ST-2258A). Typically, measurements were performed at different 25 positions on each film, and average values of *R*_s_ were calculated. Transmittance in the spectral range of 400–800 nm was measured using a Perkin-Elmer Lambda 950 UV/Vis/NIR spectrophotometer equipped with a 150-mm-diameter integrating sphere by excluding those of the substrates, and the haze was determined by the ratio of diffused to total transmittance 100(*T*_diffused_/*T*_total_). The SEM characterization and the tapping-mode AFM measurement were conducted using a Hitachi S-4800 SEM and a Veeco 3100 SPM, respectively. The TEM images were obtained with a Tecnai F20 microscope. The deposition rate and the film thickness of the deposited material were monitored by a quartz crystal oscillator mounted adjacent to the substrates.

## Additional Information

**How to cite this article:** Yang, X. *et al*. Optimizing ultrathin Ag films for high performance oxide-metal-oxide flexible transparent electrodes through surface energy modulation and template-stripping procedures. *Sci. Rep.* **7**, 44576; doi: 10.1038/srep44576 (2017).

**Publisher's note:** Springer Nature remains neutral with regard to jurisdictional claims in published maps and institutional affiliations.

## Supplementary Material

Supporting Information

## Figures and Tables

**Table 1:** Optical and electrical parameters of transmittance, sheet resistance, and FoM obtained from ITO, single Ag layer, AAM and reference in recently studies.

	ITO^46^	Ag	ZnO/AgO/ZnO^15^	ZrOx/Ag^16^	MoO_3_/Ag/MoO_3_^18^	Al_2_O_3_/Ag/MoO_x_
*T* (%)	85.7	70.79	90	79	79.73	93.85
*R*_s_ (Ω sq^−1^)	13.36	6.90	20	6.1	6.20	7.39
FoM	175	145	174	247	254	791
