# Supplementary material for: Optimizing ultrathin Ag films for high performance oxide-metal-oxide flexible transparent electrodes through surface energy modulation and template-stripping procedures
Source: Sci Rep. 2017 Mar 14;7:44576. doi: 10.1038/srep44576 (PMC5349598; doi:10.1038/srep44576)
Supplement: Supporting Information [file srep44576-s1.pdf]

Supporting Information

Optimizing ultrathin Ag films for high performance oxide-metal-oxide flexible transparent electrodes through surface energy modulation and template-stripping procedures

Xi Yang[1], Pingqi Gao[1], Zhenhai Yang[1], Juye Zhu[1], Feng Huang[1], Jichun Ye[1]

[1]Ningbo Institute of Material Technology and Engineering, Chinese Academy of Sciences, Ningbo 315201, China

e-mail: gaopingqi@nimte.ac.cn; jichun.ye@nimte.ac.cn

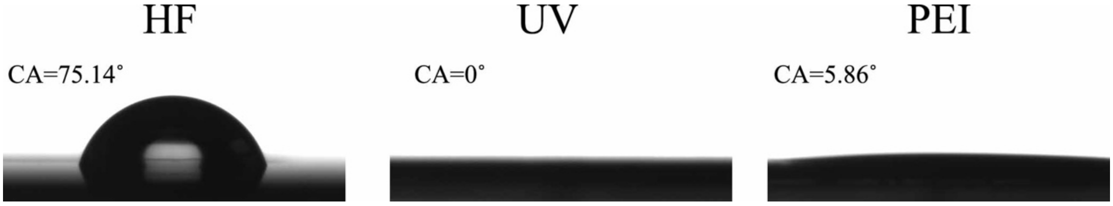

Supplementary Figure S1. Photographs of water droplets on the surfaces of substrates treated by HF, UV and PEI, respectively.

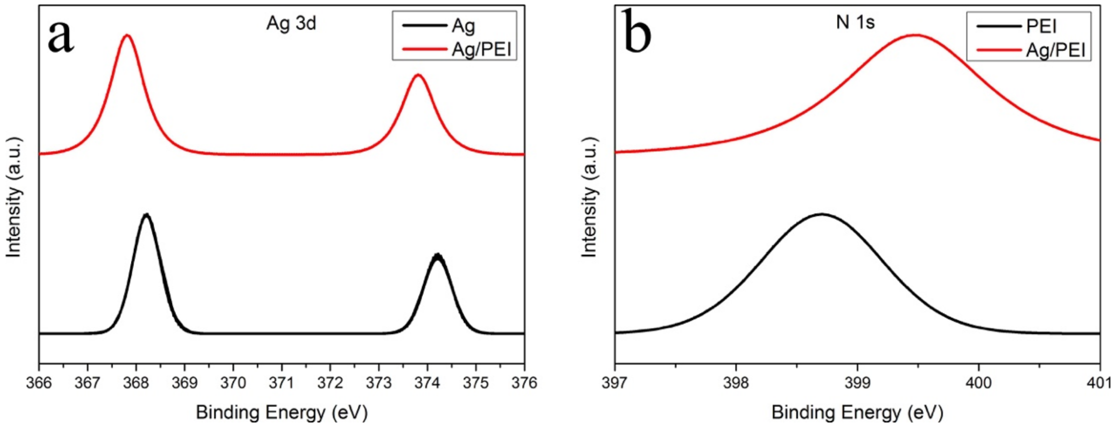

Supplementary Figure S2. High-resolution XPS data of the bare-Ag, Si substrate immediately after PEI treatment, and Ag coated on PEI treated Si (Ag/PEI) samples: **(a)** Ag 3d, and **(b)** N 1s.

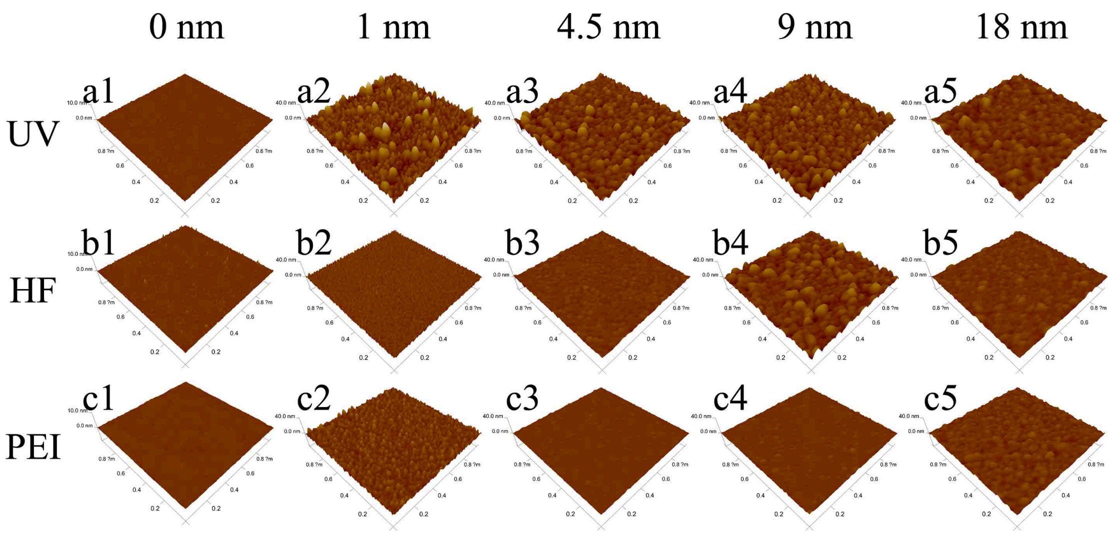

Supplementary Figure S3. 3-dimensional AFM images of various thickness of Ag on substrates treated by **(a1) - (a5)** UV, **(b1) - (b5)** HF and **(c1) - (c5)** PEI, respectively.

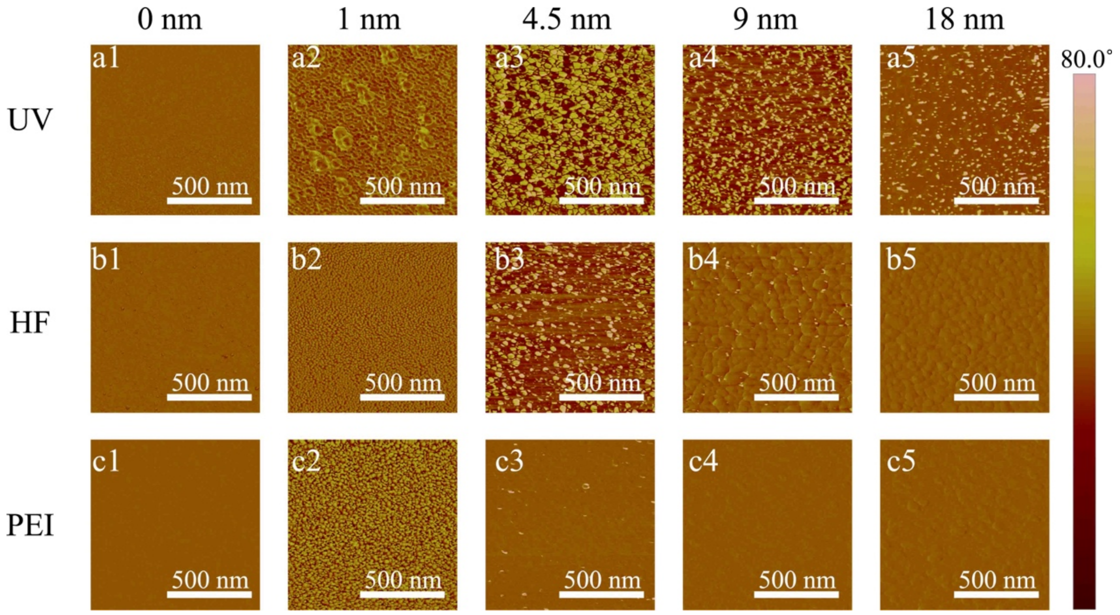

Supplementary Figure S4. AFM phase images of various thickness of Ag on substrates treated by **(a1) - (a5)** UV, **(b1) - (b5)** HF and **(c1) - (c5)** PEI, respectively.

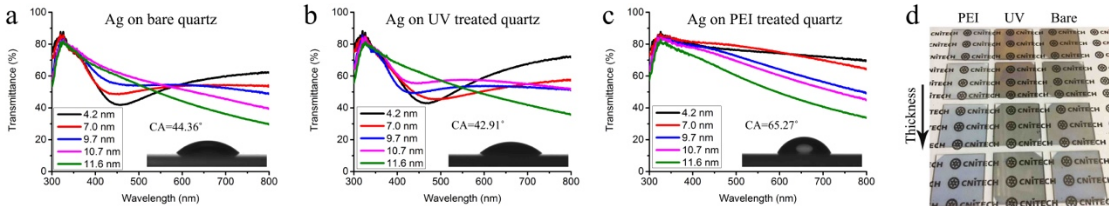

Supplementary Figure S5. (a)-(c) Optical transmittance spectra and **(d)** photographs of various thickness of Ag film on quartz substrates that were pre-treated by different procedures. The inset depicts photograph of water droplet on the surface of substrate.

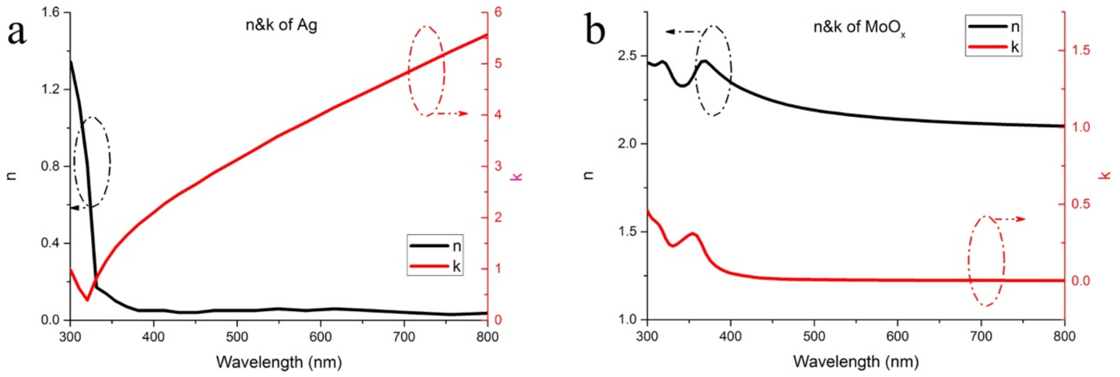

Supplementary Figure S6. Refractive indexes of **(a)** Ag and **(b)** MoO_x used in simulation.

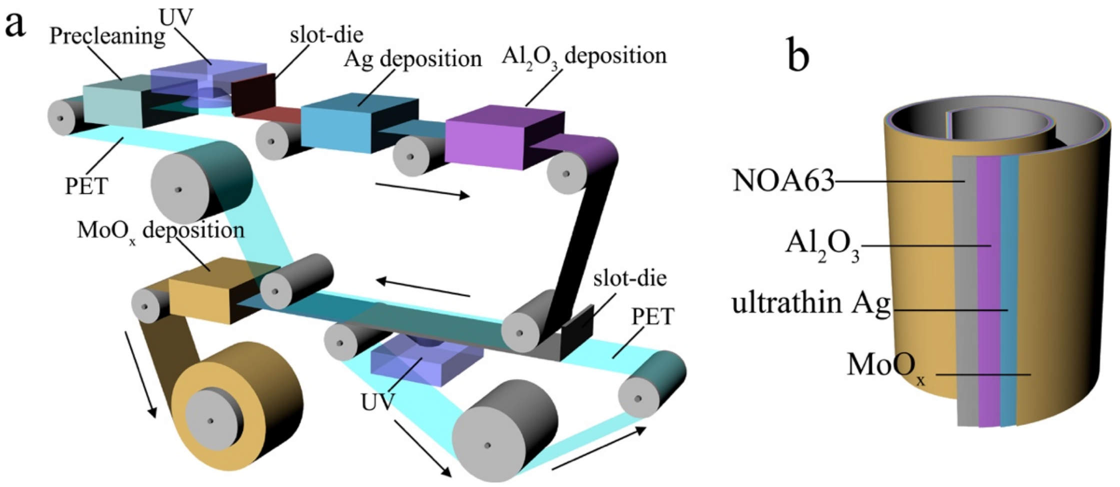

Supplementary Figure S7. Diagrams for the fabrication process of the large-area AAM based TCEs. **(a)** Continuous roll-to-roll sputtering system to fabricated AAM based TCEs. **(b)** Structure of the fabricated AAM based TCEs.

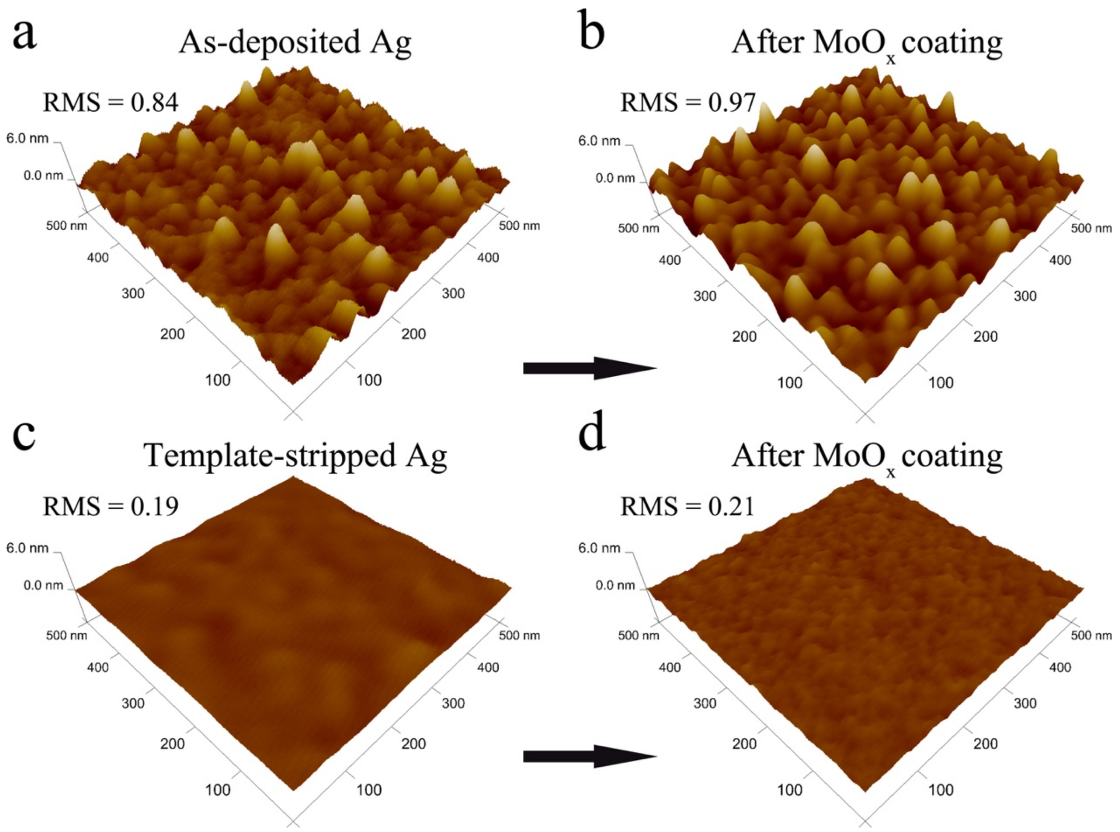

Supplementary Figure S8. AFM topology images of of as-deposited and template-stripped Ag film before and after MoO_x layer coating.

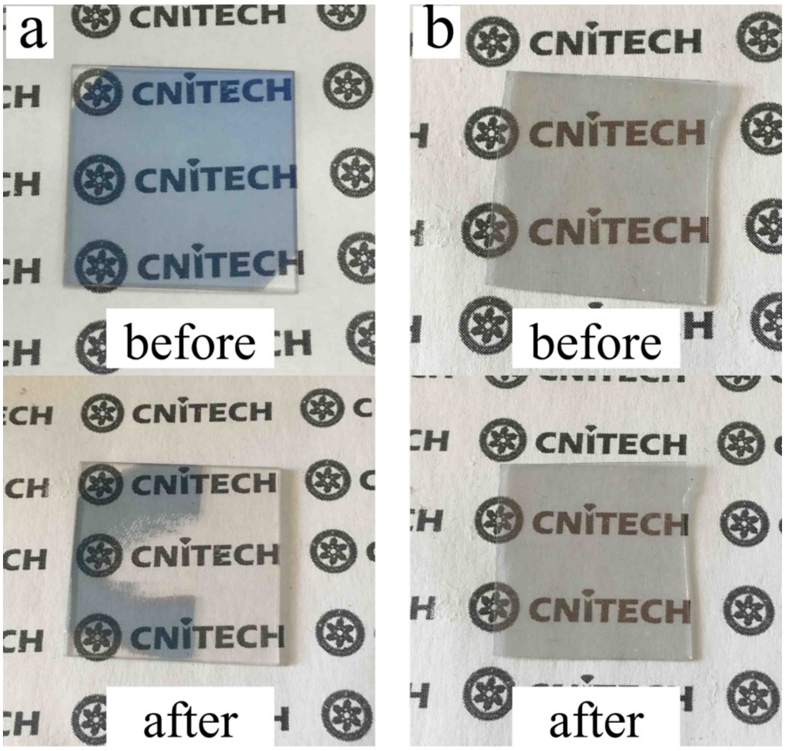

Supplementary Figure S9. Photographs of **(a)** as-deposited ultrathin Ag film and **(b)** template-stripped Ag film before (up) and after (down) the scraping test.

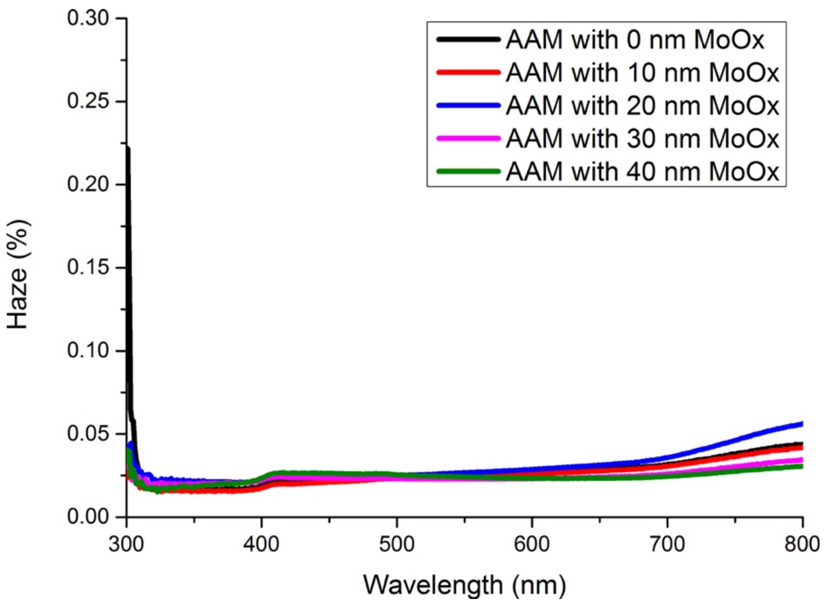

Supplementary Figure S10. The haze curves of the AAM films with various thickness of MoO_x layer.
